# Disagreements in Medical Ethics Question Answering Between Large Language Models and Physicians

**DOI:** 10.21203/rs.3.rs-5382879/v1

**Published:** 2024-11-15

**Authors:** Shelly Soffer, Dafna Nesselroth, Keren Pragier, Roi Anteby, Donald Apakama, Emma Holmes, Ashwin Shreekant Sawant, Ethan Abbott, Lauren Alyse Lepow, Ishita Vasudev, Joshua Lampert, Moran Gendler, Nir Horesh, Orly Efros, Benjamin S Glicksberg, Robert Freeman, David L Reich, Alexander W Charney, Girish N Nadkarni, Eyal Klang

**Affiliations:** Rabin Medical Center; Meuhedet Health Services; Maccabi Institute for Health Services Research; Massachusetts General Hospital; Mount Sinai Hospital; Icahn School of Medicine at Mount Sinai; Icahn School of Medicine at Mount Sinai; Icahn School of Medicine at Mount Sinai; Icahn School of Medicine at Mount Sinai; Icahn School of Medicine at Mount Sinai; Icahn School of Medicine at Mount Sinai; Azrieli Faculty of Medicine; Cleveland Clinic Florida; Sheba Medical Center; Icahn School of Medicine at Mount Sinai; Icahn School of Medicine at Mount Sinai; Icahn School of Medicine at Mount Sinai; Icahn School of Medicine at Mount Sinai; Icahn School of Medicine at Mount Sinai; Icahn School of Medicine at Mount Sinai

## Abstract

**Importance.:**

Medical ethics is inherently complex, shaped by a broad spectrum of opinions, experiences, and cultural perspectives. The integration of large language models (LLMs) in healthcare is new and requires an understanding of their consistent adherence to ethical standards.

**Objective.:**

To compare the agreement rates in answering questions based on ethically ambiguous situations between three frontier LLMs (GPT-4, Gemini-pro-1.5, and Llama-3–70b) and a multi-disciplinary physician group.

**Methods.:**

In this cross-sectional study, three LLMs generated 1,248 medical ethics questions. These questions were derived based on the principles outlined in the American College of Physicians Ethics Manual. The topics spanned traditional, inclusive, interdisciplinary, and contemporary themes. Each model was then tasked in answering all generated questions. Twelve practicing physicians evaluated and responded to a randomly selected 10% subset of these questions. We compared agreement rates in question answering among the physicians, between the physicians and LLMs, and among LLMs.

**Results.:**

The models generated a total of 3,744 answers. Despite physicians perceiving the questions’ complexity as moderate, with scores between 2 and 3 on a 5-point scale, their agreement rate was only 55.9%. The agreement between physicians and LLMs was also low at 57.9%. In contrast, the agreement rate among LLMs was notably higher at 76.8% (p < 0.001), emphasizing the consistency in LLM responses compared to both physician-physician and physician-LLM agreement.

**Conclusions.:**

LLMs demonstrate higher agreement rates in ethically complex scenarios compared to physicians, suggesting their potential utility as consultants in ambiguous ethical situations. Future research should explore how LLMs can enhance consistency while adapting to the complexities of real-world ethical dilemmas.

## Introduction

Ethics in medicine, focusing on moral goodness, badness, right, and wrong ^1^, varies significantly depending on societal and contextual factors ^2–4^. While ethicists can navigate these complexities, physicians, with variable experience, face challenges in making decisions in ambiguous and intricate ethical dilemmas ^5–7^. The emergence of large language models (LLMs) like GPT-4 ^8,9^, Gemini ^10^, and Llama-3 ^11^ has revolutionized language processing and generation in medicine ^12,13^, prompting an evaluation of their performance in ethically ambiguous situations.

Empathy and ethical reasoning have traditionally been viewed as uniquely human traits ^14–16^. However, recent studies indicate that AI can mimic these attributes, delivering empathetic and ethically reasoned responses to patient inquiries ^9,17^. Systematic approaches have demonstrated that ethical reasoning can be integrated into clinical practice, raising the question of whether LLMs can be similarly trained to handle ethical issues ^18^.

This study aims to explore the consistency of LLMs in navigating ethical ambiguities compared to medical professionals. By tasking LLMs with formulating complex ethical questions and comparing the agreement rates among the models and between the models and physicians, this research seeks to evaluate the potential of LLMs as consultants in ethical decision-making within healthcare.

## METHODS

### Study Design

**Figure 1:**
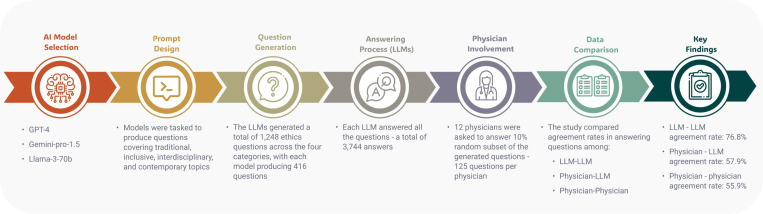
Overview of the Study Design Comparing Agreement Rates on Medical Ethics Questions Between Large Language Models and Physicians. Three large language models (LLMs) generated 1,248 ethically complex questions across four categories: traditional, interdisciplinary, inclusive, and contemporary. Each model answered all questions, resulting in 3,744 responses. Twelve physicians each answered a random 10% subset of these questions (125 responses per physician). Agreement rates were calculated for three comparisons: LLM versus LLM, physician versus physician, and physician versus LLM. The LLMs demonstrated a higher agreement rate (76.8%) compared to physician-physician (55.9%) and physician-LLM (57.9%) agreement rates.

This cross-sectional study evaluated the performance of three LLMs—GPT-4, Gemini-pro-1.5, and Llama-3–70b—in generating and responding to complex medical ethics questions. The study compared the agreement rates among these models and between the models and a group of twelve practicing physicians. The study design is illustrated in Fig. 1. The research project was approved by the Mount Sinai Institutional Review Board (IRB).

### Prompt Design for Question Creation

To generate complex ethical questions, we crafted detailed prompts for each LLM, targeting the production of ethically ambiguous, and challenging scenarios. Each prompt directed the models to create multiple-choice questions based on specific instructions. We derived topics and sub-topics from the American College of Physicians Ethics Manual ^19^, which outlines a comprehensive framework of six subjects and 52 sub-subjects (Supplementary eTable 1).

We targeted four categories—traditional, inclusive, interdisciplinary, and contemporary—to address a broad spectrum of issues in medical ethics and professionalism ^2,19^. We refined each prompt through iterative engagements with the three LLMs, ensuring the generation of questions that were both ethically ambiguous and complex.

Following the prompt design, each LLM was tasked with generating two questions for each of the four categories across all 52 sub-subjects outlined in the American College of Physicians Ethics Manual. This approach resulted in the production of 416 questions per model.

The complete prompt with the JSON directive and formatting is in Supplementary eFigure 1.

### Prompt Design for Answering and Reasoning

To assess LLMs’ capacity to analyze and respond to the generated ethical questions, we presented them with a second prompt. This prompt required the models to answer the questions, articulate their reasoning, and identify the ethical principles guiding their responses. Additionally, the LLMs evaluated each question on dimensions of complexity, novelty, inclusiveness, significance, and relevancy, and categorized each by medical topic. The complete font with the JSON directive and formatting is presented in Supplementary eFigure 2.

**Figure 2:**
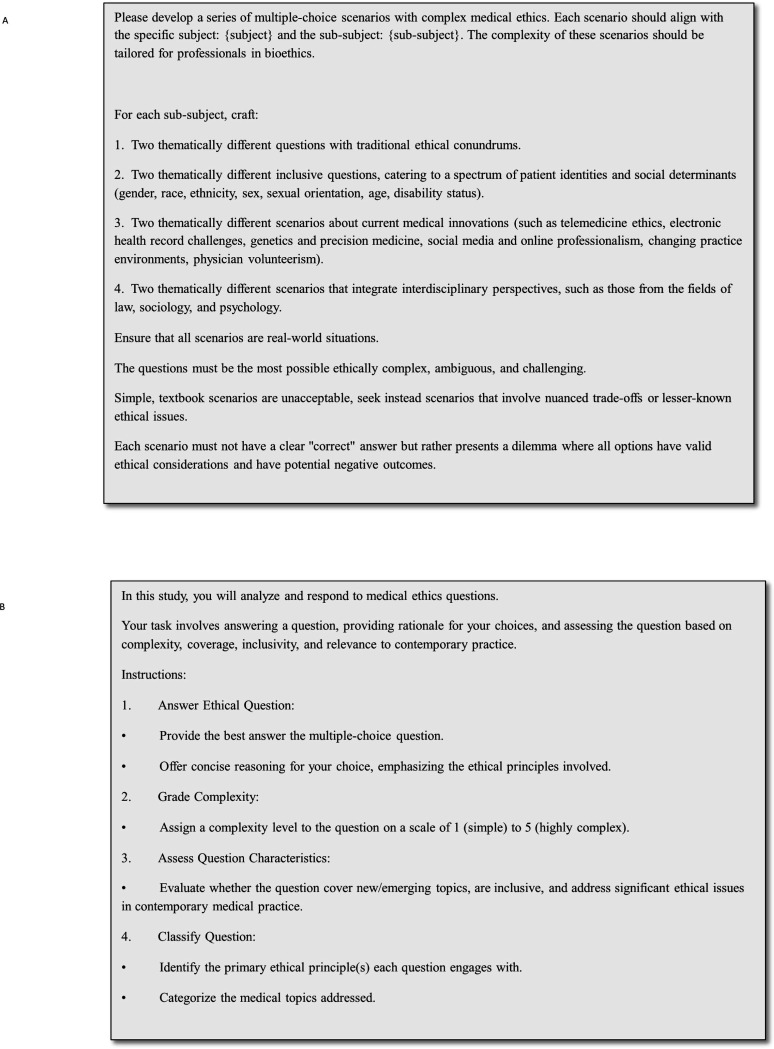
**(A)** Prompt for question creation and **(B)** prompt for answering and reasoning

The final version of the prompt used for question generation and the prompt used for answering and reasoning are displayed in Fig. 2A–B.

### Analysis on Agreement Rates

We analyzed responses from each LLM to questions they generated as well as to those generated by other models (LLM-LLM). We specifically examined the extent of agreement, comparing responses across different subjects, sub-subjects, and question types. LLM-LLM agreement calculation was limited to each LLM answering the other two LLMs questions.

### Human Comparison

To compare the ethical reasoning of the models with that of human experts, 12 physicians independently answered a random 10% sample of the questions generated by each model (amounting to 125 questions per physician). To prevent bias, the physicians were blinded to the identity of the LLMs. We examined the level of agreement among the physicians themselves (physician-physician) as well as the agreement between the physicians’ answers and those provided by the models (physician-LLM). Additionally, the physicians classified the questions for complexity, novelty, relevance, significance and inclusiveness.

The physicians specialize in various medical fields and come from different backgrounds and countries (Supplementary eTable 2). The prompt for the physicians is presented in the Supplementary eFigure 3.

### Experimental Setup

We conducted GPT-4 experiments through the Mount Sinai Hospital (MSH) Azure tenant API using gpt-4–0125-preview. We conducted Gemini experiments through Google’s API setup using Gemini-pro-1.5 version. In contrast, inferences for Llama-3 were performed on a local cluster equipped with 4xH100 GPUs, utilizing Llama-3–70b-instruct 16-bit version. We used default hyper-parameters (temperature, top-k, max-length, etc.) for all models. We utilized Python (3.10) for data analyses. We used several Python libraries to facilitate data processing, model interaction, and analysis: NumPy (1.26.4) for numerical computations, Pandas (2.1.4) for data manipulation, Scikit-Learn (1.3.0) for statistical analysis, Hugging Face’s Transformers (4.37.2) and torch (2.2.2 + cu121) for accessing pre-trained NLP models, and the json module (2.0.9) for handling JSON data formats.

### Statistical and Error Analysis

For the analysis of binary data, Chi-square tests were used to compare response frequencies. Ordinal data were examined using Kruskal-Wallis tests. P-values less than 0.05 were considered statistically significant. We conducted a qualitative error analysis to pinpoint the sources of disagreement among the models and among physicians. This involved a review of conflicting responses, categorizing the underlying reasons for these discrepancies. Our examination focused on ambiguity, oversimplification of complex issues, and lack of contextual detail in generalizations. We further explored how each model prioritized different ethical principles and identified patterns of disagreement across specific sub-subjects and question types. This analysis elucidated key factors contributing to the inconsistencies observed in ethical reasoning.

## RESULTS

**Figure 3:**
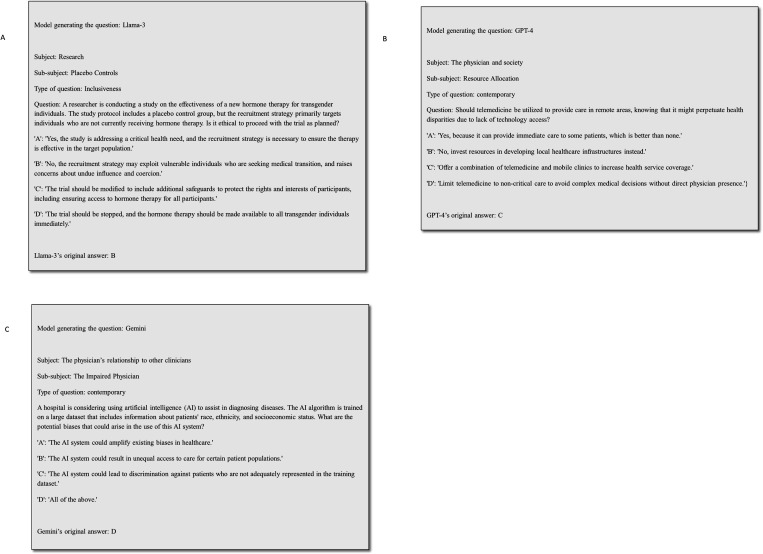
**A-C.** Examples of generated questions and the model’s answers

Each model produced a distinct set of ethical questions, resulting in a total of 1248 questions across all models (416 for each model). These questions were answered by the model and then cross-evaluated, with each model answering questions generated by itself and the other models (a total of 3744 answers). Figures 3A–C present examples of generated questions and the model’s answers. A table with all questions and answers by the different models is provided in eTable 2.

### Analysis of Consensus in Models’ Responses

**Figure 4:**
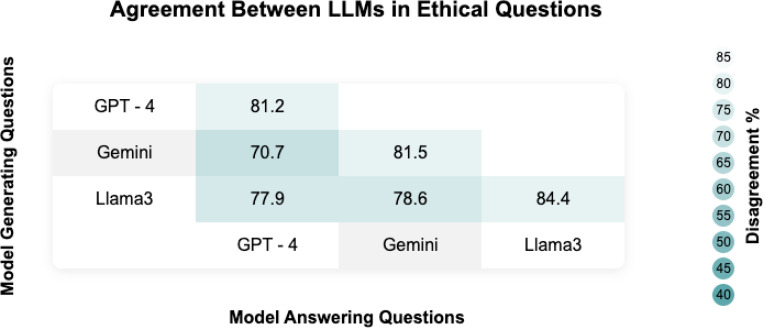
Heatmap describing the agreement across models.

Model agreements in answering ethical questions are presented in Fig. 4 and Supplementary eTable 3. Agreement scores ranged from 70.7% for GPT-4 answering Gemini-generated questions to 84.4% for Llama-3 answering its own questions. Each model generally demonstrated higher agreement when answering its own questions, with GPT-4 at 81.2%, Gemini at 81.5%, and Llama-3 at 84.4%. However, cross-model agreements were lower, such as 70.7% for GPT-4 on Gemini questions and 75.0% for Gemini on GPT-4 questions. The overall LLM-LLM agreement rate (for each LLM answering other LLMs questions) was 76.8%.

### Models’ Agreement by Subject, Sub-subject, and Type

Supplementary eTable 4 presents the percentage of agreement across various subjects. Agreement ranged from 83.9% in “The Physician’s Relationship to Other Clinicians” to 71.1% in “The Ethics of Practice” (p = 0.003).

Supplementary eTable 5 illustrates the percentage of agreement among LLMs across various medical ethical sub-subjects. The lowest agreement was observed in “Placebo Controls” at 55.6%, followed by “Conflicts of Interest” at 61.1%, and “Informed Decision Making and Consent” as well as “Physician-Assisted Suicide and Euthanasia,” both at 63.9%. Conversely, the sub-subjects with the highest agreement included “Decisions About Reproduction” at 91.7% and “Expert Witnesses” at 93.1%. The agreements across different question types are presented in Supplementary eTable 6.

### Analysis of Question Characteristics

All models classified the questions as inclusive and significant (Supplementary eTable 7). The complexity and relevance grades given by the models are presented in Supplementary eTable 8.

### Analysis of Ethical Principles and Reasoning

The analysis conducted by the models revealed a high level of agreement on key ethical principles. Beneficence, autonomy, justice, and non-maleficence were the most prominent principles identified by the models. Other frequently cited principles included confidentiality, respect for persons, professionalism, privacy, informed consent, and transparency. Supplement eFigure 4A–C show co-occurrence graphs that present the accordance of the top ten frequent principles for each model.

### Classification of Questions by Medical Topics

Each model tagged the questions to a specific group, which were then grouped into 10 broad categories. The categories and their frequency are presented in the Supplementary eTable 9. Agreement proportions did not differ between the groups.

Of note, some specific topics were recurrent, with transgender issues being particularly prominent accounting for approximately 35% of the inclusiveness questions. The GPT-4 model generated the most questions related to transgender topics, with a total of 41 unique questions, followed by the Gemini model with 35 questions and the Llama-3 model with 31 questions.

### Physician Assessment of Model-Generated Questions

The novelty, inclusiveness, significance, complexity, and relevance grades rated by physicians across the different models are detailed in Table 1. All models produced questions with high significance (over 90%) and moderate complexity (scores between 2 and 3 out of 5). About half of the questions were novel, and roughly 60% were rated as inclusive.

### Analysis of Physician Consensus and Physician-Model Agreement

**Figure 5:**
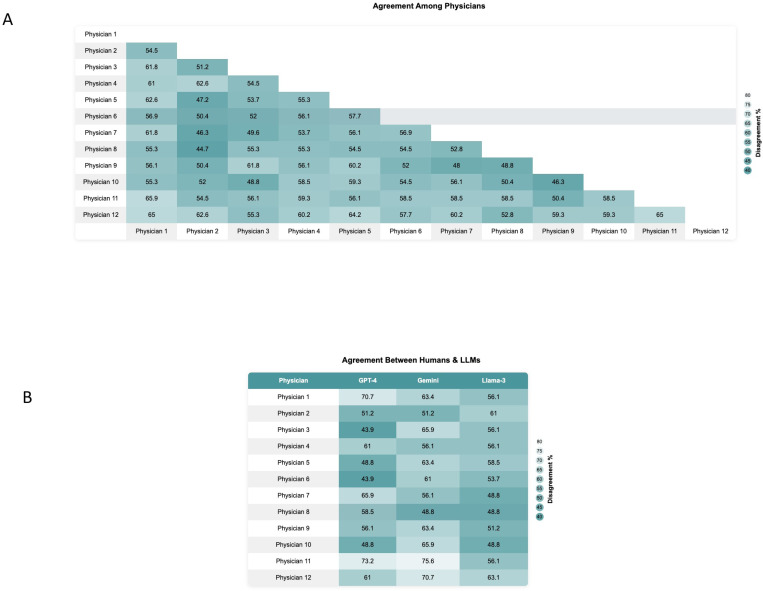
**(A)** Heatmap illustrating the agreement among physicians regarding their responses to the LLMs medical ethical questions. **(B)** Heatmap demonstrating the agreement between individual physicians and the three different LLMs: GPT-4, Gemini, and Llama-3.

Physician-physician agreement was significantly lower than LLM-LLM agreement (55.9% vs. 76.8%, p < 0.001), highlighting considerable variability among human physicians. Figure 5A presents a heatmap of the percentage of agreement among physicians.

The overall physician-LLM agreement (57.9%) was also significantly lower than LLM-LLM agreement (76.8%) (p < 0.001). Figure 5B illustrates the agreement levels between each physician and the three models.

The overall physician-LLMs agreement rates across different models ranged from 54.9% (physician-Llama-3) to 61.8% (physician-Gemini), with no statistically significant differences among the models (Supplementary eTable 10).

A table with the questions and answers by all physicians is provided in Supplementary Excel File 2.

### Error Analysis and Discrepancy Evaluation

Several factors contributed to the observed disagreements among the models and physicians:

1. *Ambiguity and lack of prioritization*: Many disagreements arose when reasoning included multiple ethical principles without clearly indicating how to balance them. For example, GPT-4 often incorporated a broad range of principles, including equity and patient-centered care, but struggled to prioritize these when they conflicted with each other.
2. *Oversimplification of complex issues*: Simplifying complex ethical dilemmas without considering all relevant factors and principles led to differing interpretations. Llama-3, for instance, tended to emphasize cultural sensitivity and respect for autonomy, which sometimes oversimplified the nuanced requirements of specific medical contexts.
3. *Generalization*: Providing general reasoning without addressing specific contextual details and nuances resulted in varied conclusions. Gemini frequently emphasized traditional principles like autonomy and non-maleficence without sufficiently considering the unique aspects of each scenario.

Specific sub-subjects with high disagreement rates included “Conflicts of Interest” (Examples: Supplementary Excel File 1; rows 1068, 2318), “Informed Decision Making and Consent” (Examples: Supplementary Excel File 1; rows 2954, 2955), “Sexual Contact Between Physician and Patient” (Examples: Supplementary Excel File 1; row 3024), and “Physician-Assisted Suicide and Euthanasia” (Examples: Supplementary Excel File 1; rows 613, 3529).

Interdisciplinary (n = 90) and contemporary (n = 78) questions exhibited the highest rates of disagreement among models. For example, GPT-4’s diverse topic range, including transgender health and cognitive impairments, often led to differing ethical interpretations compared to Gemini’s more traditional focus and Llama-3’s culturally sensitive approach (Examples: Supplementary Excel File 1; rows 1541, 458). Similarly, human physicians showed varied interpretations on interdisciplinary and contemporary issues, such as collaboration with social workers on patient care plans, involving legal advisors in decisions for patients with diminished capacity, and areas like genetic testing and its impact on patient privacy (Examples: Supplementary Excel File 1; row 2919).

Another recurring pattern of disagreement involved scenarios where physicians consulted ethics committees, legal advisors, or peers versus making decisions independently (Examples: Supplementary Excel File 1; rows 3, 19, 241, 458, 896). In cases like a Jehovah’s Witness refusing a blood transfusion or disclosing a pregnancy, models varied between respecting patient autonomy and seeking external intervention. This included actions such as contacting the courts to request a legal order or informing child protective services.

The analysis of the dataset reveals instances where there was a consensus among LLMs on the correct answers, yet significant disagreement persisted among humans. For example, in questions addressing patient autonomy and end-of-life care, the LLMs uniformly selected answers that aligned with established ethical principles such as autonomy and beneficence. However, human experts exhibited divergent views, reflecting their nuanced interpretations and the influence of personal, cultural, and professional experiences on ethical decision-making (Examples: Supplementary Excel File 1; rows 550, 840). Another notable example of the divergence between LLMs and human arises in the context of medical confidentiality. In scenarios where LLMs uniformly agreed on the ethical course of action—such as maintaining strict confidentiality in cases involving adolescent patients seeking advice on sexual health—human experts displayed a range of opinions (Examples: Supplementary Excel File 1; row 435). Some experts argued for exceptions to confidentiality based on the potential risks to the patient or others, while others adhered strictly to the principle of patient autonomy.

## DISCUSSION

LLMs, such as GPT-4, Gemini-pro-1.5, and Llama-3–70b, demonstrated higher agreement rates in ethically ambiguous situations compared to physicians. Specifically, the agreement rates among LLMs were significantly higher (76.8%) than those among physicians (55.9%), and the between LLMs and physicians (57.9%). This suggests that LLMs adhere more consistently to established ethical principles like beneficence and autonomy, whereas physicians exhibit a broader range of interpretations influenced by personal, cultural, and professional backgrounds.

Previous research supports the notion that LLMs can provide ethical guidance that is perceived as highly virtuous, intelligent, and trustworthy, and in some cases, superior to humans ^9,20–22^. Our study extends this understanding by evaluating the ability of LLMs to generate and respond to complex ethical dilemmas consistent with the American College of Physicians Ethics Manual ^19^. The high significance ratings above 90%, novelty ratings of about 50%, and inclusiveness ratings of approximately 60%, underscore LLMs ability to produce pertinent and thought-provoking content. Although complexity scores were judged as moderate by humans, this contrasts with the low agreement rate among physicians on answers, which suggests non-trivial questions.

Rao et al. argue against aligning LLMs with specific ethical principles, advocating instead for the infusion of generic ethical reasoning capabilities ^21^. They found that while LLMs like GPT-4 can demonstrate nearly perfect ethical reasoning, they still exhibit biases towards Western values. Beneficence, autonomy, justice, and non-maleficence, the principles predominantly identified by the models’ reasoning, are emphasized in Western bioethics, particularly through principlism, which is prevalent in Western medical ethics education and practice ^5^. Due to the lack of transparency in LLMs, understanding their ethical decision-making process and their ability to account for diverse cultural nuances remains a significant challenge.

The comparison with human experts underscored the intricacies of ethical decision-making. Practicing physicians, diverse in cultural, demographics, disciplines, and training backgrounds, regularly confront complex medical ethics issues. In our study, disagreement among 12 physicians were significant, above 40%, paralleling the high discord observed between the models and the physicians. This indicates that ethical reasoning in medical contexts is profoundly subjective, influenced by cultural and personal factors and biases ^23,24^. Such variability among physicians highlights the complex nature of medical ethics and the inherent difficulties in reaching a consensus.

The better uniformity in algorithmic responses, compared to the diverse human perspectives, suggests that algorithms may excel in capturing the deep context embedded by ethical thought leaders. This consideration invites a nuanced reflection: if ethical principles, articulated by experts, are open to interpretation, could algorithms not serve as accurate consultants? By being meticulously context-aware and with careful training to reduce bias, algorithms could potentially offer consistent consultations on established ethical frameworks. This is not to claim brilliance on the part of the algorithms, but rather to highlight their capacity for accurate and considered interpretation, qualities that are especially valuable in the high-stakes, variably interpreted realm of medical ethics.

Conversely, the disagreement between humans might emphasize the concept of *value pluralism*, which acknowledges that multiple, sometimes conflicting, values can be equally correct and fundamental ^4,25^. This perspective is crucial in medical ethics, where decisions often involve balancing diverse and competing values. Even if LLMs can create *ethical consistency* using the knowledge of ethics experts, it does not prevent the possibility of moral dilemmas, as sometimes multiple actions that ought to be done cannot be accomplished simultaneously ^26^. Our study highlights the importance of equipping LLMs with the ability to navigate this complexity, ensuring they can handle a variety of ethical frameworks and cultural contexts without defaulting to a single moral stance.

This study has several limitations. Firstly, we used only three LLMs, whereas many more are available, potentially offering diverse insights. Secondly, the multiple-choice question format may not fully capture the intricacies and nuances of real-world ethical practice. Furthermore, the ethical scenarios we presented were hypothetical, potentially lacking the dynamic nature of medical situations. This limitation is critical as the high disagreement rate among humans in these simplified scenarios suggests that real- world complexities could pose even greater challenges for physicians. Our focus was on generating and responding to questions rather than engaging in continuous ethical discourse, which might limit the depth of ethical analysis. Additionally, our sample size was limited to 12 physicians, which does not adequately represent the global medical community’s diversity. Moreover, we limited the exploration to default hyper-parameters set in the models’ API calls. We also did not experiment with techniques such as fine-tuning or retrieval augmented generation (RAG), opting instead to consider “out-of-the-box” performance ^13,27^. Lastly, the evaluation of responses relied on a diverse group of physicians, and not ethical experts – however, this represents real-world setting, where most physicians are not ethical experts.

In conclusion, LLMs exhibit significantly higher in-silico agreement and consistency rates as those of physicians in difficult ethical dilemmas, suggesting their potential as consultants in ambiguous ethical scenarios. However, the value pluralism inherent in human decisions, which often balance diverse and conflicting stances, presents a challenge. While LLMs can streamline ethical consistency, they must also adapt to the complexities of moral dilemmas involving multiple valid actions. This juxtaposition invites careful consideration and ongoing dialogue about how technology might enhance, yet respect, the multifaceted nature of medical ethics.

## Supplementary Material

Supplement 1

## Figures and Tables

**Table 1:** Novelty, inclusiveness, significance, complexity and relevance grades rated by physicians across the different models

Metrics	GPT-4	Gemini	Llama-3	P value
Significance	452/492 (91.9%)	461/492 (93.7%)	461/492 (93.7%)	0.454
Complexity	3.0 (2.0–4.0)	2.0 (1.0–3.0)	3.0 (2.0–4.0)	< 0.001
Novelty	240/492 (48.8%)	202/492 (41.1%)	248/492 (50.4%)	< 0.006
Inclusiveness	313/492 (63.6%)	297/492 (60.4%)	283/492 (57.5%)	0.132
Relevance	3.0 (2.0–4.0)	3.0 (2.0–4.0)	3.0 (2.0–4.0)	0.012

## Data Availability

Data is provided within the manuscript or supplementary information files
