# Supplementary material for: Disagreements in Medical Ethics Question Answering Between Large Language Models and Physicians
Source: Res Sq. 2024 Nov 15:rs.3.rs-5382879. Preprint. [Version 1] doi: 10.21203/rs.3.rs-5382879/v1 (PMC11601831; doi:10.21203/rs.3.rs-5382879/v1)
Supplement: Supplement 1 [file NIHPPRS5382879V1-supplement-1.pdf]

Supplementary Files

This is a list of supplementary files associated with this preprint. Click to download.

- SupplementaryFile1.xlsx
- SupplementaryFile2.xlsx
- SupplementaryOnlinecontant.docx
